# Accurate Classification of Non-small Cell Lung Cancer (NSCLC) Pathology and Mapping of *EGFR* Mutation Spatial Distribution by Ambient Mass Spectrometry Imaging

**DOI:** 10.3389/fonc.2019.00804

**Published:** 2019-08-28

**Authors:** Min Zhang, Jiuming He, Tiegang Li, Haixu Hu, Xiaofei Li, Hao Xing, Jun Wang, Fan Yang, Qunfeng Ma, Bing Liu, Chuanhao Tang, Zeper Abliz, Xiaoqing Liu

**Affiliations:** ^1^Academy of Military Medical Science, Beijing, China; ^2^Department of Lung Cancer, The Fifth Medical Center of Chinese PLA General Hospital, Beijing, China; ^3^State Key Laboratory of Bioactive Substance and Function of Natural Medicines, Institute of Materia Medica, Peking Union Medical College, Chinese Academy of Medical Sciences, Beijing, China; ^4^Laboratory of Oncology, The Fifth Medical Center of Chinese PLA General Hospital, Beijing, China; ^5^Department of Thoracic Surgery, Tangdu Hospital, Air Force Military Medical University, Xi'an, China; ^6^Department of Thoracic Surgery, Peking University People's Hospital, Beijing, China; ^7^Department of Thoracic Surgery, The Fifth Medical Center of Chinese PLA General Hospital, Beijing, China; ^8^Department of Oncology, Peking University International Hospital, Beijing, China; ^9^Center for Imaging and Systems Biology, Minzu University of China, Beijing, China

**Keywords:** tumor heterogeneity, mass spectrometry imaging, lipids, non-small cell lung cancer (NSCLC), epidermal growth factor receptor (EGFR)

## Abstract

**Objectives:** Tumor pathology examination especially epidermal growth factor receptor (*EGFR*) mutations molecular testing has been integral part of lung cancer clinical practices. However, the *EGFR* mutations spatial distribution characteristics remains poorly investigated, which is critical to tumor heterogeneity analysis and precision diagnosis. Here, we conducted an exploratory study for label-free lung cancer pathology diagnosis and mapping of *EGFR* mutation spatial distribution using ambient mass spectrometry imaging (MSI).

**Materials and Methods:** MSI analysis were performed in 55 post-operative non-small cell lung cancer (NSCLC) tumor and paired normal tissues to distinguish tumor from normal and classify pathology. We then compared diagnostic sensitivity of MSI and ADx-amplification refractory mutation system (ARMS) for the detection of *EGFR* mutation in pathological confirmed lung adenocarcinoma (AC) and explored *EGFR* mutations associated biomarkers to depict *EGFR* spatial distribution base on ambient MSI.

**Results:** Of 55 pathological confirmed NSCLC, MSI achieved a diagnostic sensitivity of 85.2% (23/27) and 82.1% (23/28) for AC and squamous cell carcinoma (SCC), respectively. Among 27 AC, there were 17 *EGFR-*wild-type and 10 *EGFR-*mutated-positive samples detected by ARMS, and MSI achieved a diagnostic sensitivity of 82.3% (14/17) and 80% (8/10) for these two groups. Several phospholipids were specially enriched in AC compared with SCC tissues, with the higher ions intensity of phospholipids in *EGFR-*mutated-positive compared with *EGFR-*wild-type AC tissues. We also found *EGFR* mutations distribution was heterogeneous in different regions of same tumor by multi-regions ARMS detection, and only the regions with higher ions intensity of phospholipids were *EGFR-*mutated-positive.

**Conclusion:** MSI method could accurately distinguish tumor pathology and subtypes, and phospholipids were reliable *EGFR* mutations associated biomarkers, phospholipids imaging could intuitively visualize *EGFR* mutations spatial distribution, may facilitate our understanding of tumor heterogeneity.

## Highlights

- Lung tumors are heterogeneous, making diagnosis, and treatment difficult.
- Metabolomics can be used to separate lung tumor and healthy tissue in real time.
- Metabolite profiling can assess lung tumor pathology and EGFR mutation status.
- Mass spectrometry imaging analysis can visually plot the spatial distribution heterogeneity of EGFR mutation status, which may benefit our understanding of tumor heterogeneity.

## Introduction

Lung cancer is a highly heterogeneous malignant epithelial tumor with distinct pathological features and clinical behavior (1). It can be routinely classified into non-small cell lung cancer (NSCLC) and small cell lung cancer according to histopathology, with NSCLC accounts for 85% of cases. The clinical treatment program decisions in lung cancer always depended largely on accurate classification of tumor types and subtypes. Advance in molecular diagnostic technology and deep study of tumor biology, NSCLC are found to have diverse molecular subtypes according to lung cancer-specific driver oncogenes (2–5). Epidermal growth factor receptor *(EGFR)* (6–10) is one of the most common driver oncogenes, but also an important therapeutic target and good predictor of the curative effect of targeted drugs in NSCLC. Hence, accurate molecular pathology testing especially *EGFR* mutations detection has been an expert consensus in lung cancer clinical practice (11).

Tumor spatiotemporal heterogeneity remains the main reason of anti-tumor therapy failure, which has significant influence on the treatment decision making and patients' prognosis. Because of tumor spatiotemporal heterogeneity, NSCLC patients harbor *EGFR* mutations will have different drug response and clinical benefit treated with *EGFR*-tyrosine kinase inhibitors (TKIs) (1, 12–17). However, routine driver oncogenes detection methods (18, 19) including direct sequencing and ARMS have inherent limitations: lose all spatial distribution information of *EGFR* mutations during the tumor tissue homogenization process. Hence, the current gene mutation detection methods are unable to reveal the *EGFR* mutation spatial distribution features. New methods are urgently needed to intuitively visualize the spatial distribution of *EGFR* mutations across whole tumor tissues and facilitate more accurate *EGFR* mutations detection.

MSI, a spatially resolved label-free bioanalytical technique (20–25), can directly map the spatial distribution of chemical molecules (i.e., proteins and metabolites) in biological tissues, has been widely used for biomarkers screening and disease diagnosis. Air flow assisted desorption electrospray ionization-MSI (AFADESI-MSI) (20, 21) is an ambient MSI technique which specially characterized the endogenous metabolites such as lipids in biological tissues. This approach allows rapid and nearly real-time analysis with minimal pre-treatment, usually a single AFADESI-MSI analysis of a tissue section requires only tens of minutes. AFADESI-MSI can produce a multicolor map to illustrate the spatial distribution of the molecules of interest or candidate biomarkers while maintain tissue morphology integrity, will facilitate studies of tumor spatial heterogeneity.

The present study is an extension of our previous work (20) with the goal to explore *EGFR* mutations associated biomarkers, and visualize *EGFR* mutation spatial distribution in lung adenocarcinoma (LADC) tissues using ambient AFADESI-MSI.

## Materials and Methods

### Sample Collection and Pre-treatment

All post-operative lung cancer tissue and paired adjacent normal (more than 5 cm to tumor) samples were collected from the Tang Du Hospital of Air Force Military Medical University, Peking University People's Hospital, and the Fifth Medical Center of Chinese PLA General Hospital following ethics committee approval number: 2012-11-171. The enrolled patients were all newly diagnosed did not show other tumor occurrences. Patients had not received chemotherapy or radiotherapy prior to surgery. Study protocols were approved by the ethical review community of the Fifth Medical Center of Chinese PLA General Hospital, and all study participants provided informed written consent. Samples were washed twice with sterile saline to remove the blood clots. Then the samples were flash frozen in liquid nitrogen and stored at −80°C before being sectioned at 8-μm thickness using a cryomicrotome (CM 1950; Leica, Wetzlar, Germany) and thaw-mounted onto glass slides (Superfrost Plus slides, Thermo Fisher Scientific, Waltham, MA, USA). Five tissue sections were cut and collected, and one cryosection was acetone-fixed and subsequently stained using haematoxylin and eosin (H&E) for pathological examination. Other slides were stored in closed containers at −80°C for AFADESI-MSI analysis. Prior to analysis, the slides were allowed to thaw at room temperature and were dried in a desiccator for ~30 min.

### Histopathology Analysis and *EGFR* Mutation Detection

Tumor content and pathology was assessed by two independent pathologists according to H&E-stained and immunohistochemistry (streptavidin-perosidase, S-P) examination. Detection of *EGFR* mutations was undertaken using an ADx-ARMS *EGFR* mutation test kit (Amoy Diagnostics, Xiamen, China) according to the manufacturer's instructions on a 7500 real-time PCR System (Applied Biosystems, Foster City, CA, USA). DNA quality and integrity were evaluated according to the 260 nm/280 nm ratio using a NanoDrop 2000 Ultramicro spectrophotometer (Thermo Fisher Scientific). We defined the presence of an *EGFR* mutation as Ct <26 (representing the number of cycles in which the fluorescent signal from each reaction tube reached the set threshold) and mutation abundance >5%.

### MSI Profiling and Metabolite Identification

The MSI profiling experiments were performed using a Q-TOF (QSTAR Elite, Applied Biosystem/MDS Sciex) equipped with a custom-made AFAI ion source as previously described (20). Metabolite assignments were tentatively confirmed by tandem mass spectrometry (MS/MS) based on a liquid chromatography mass spectrometry (LC-MS) technique (26–28). Briefly, frozen lung tissue samples (wet weight: ~50 mg) were homogenized in a pre-cooled solution (410 μL of methanol and 210 μL of water), followed by addition of 280 μL of methylene dichloride and 210 μL of water. Homogenates were thoroughly vortexed for 2 min, followed by centrifugation at 15,000 rpm for 10 min at 4°C. Centrifugation produced a biphasic mixture, with the upper (polar) and lower (non-polar) layers collected separately and dried under nitrogen. Before analysis, polar extracts were resuspended in 1.2 mL of 8:2 acetonitrile: water, and non-polar extracts were resuspended in 120 μL of 40:60 acetonitrile: water. Analyses were conducted on an UltiMate 3000 RSLC System (Dionex; Thermo Fisher Scientific) coupled to a Q-Orbitrap mass spectrometer (Q-Exactive; Thermo Fisher Scientific). The MS spray voltage was ± 3.5 kV, and the capillary temperature was set to 350°C, with the sheath gas at 40 arbitrary units and the aux gas at 11 arbitrary units. The chromatographic peak width was 15 s, and the mass scan range was set to a range of 70 Da to 1,000 Da. The resolution of the Orbitrap was set to 70,000. MS/MS data were collected with the collision energy between ± 10 and ± 45 eV. A linear 30-min water (5 mM ammonium acetate)-to-acetonitrile gradient was run on a Phenomenex Kinetex HILIC column (2.6 μm, 2.1 × 150 mm; Phenomenex, Torrance, CA, USA) for the polar sample. For the non-polar layer, a linear 35-min water (0.1% formic acid)-to-acetonitrile gradient was employed on a Waters ACQUITY UPLC CSH C18 column (1.7 μm, 2.1 × 100 mm; Waters Corp., Milford, MA, USA). Data regarding exact molecular weights (accurate masses of parent and product ions in MS/MS spectra) retrieved from the literature or online databases [HMDB (http://www.hmdb.ca/metabolites), Massbank (https://massbank.eu/MassBank/Index), and Lipid maps (http://www.lipidmaps.org/)] were used to tentatively confirm the postulated structures of these potential biomarkers.

### Data Processing and Statistical Analysis

**Figure 1:**
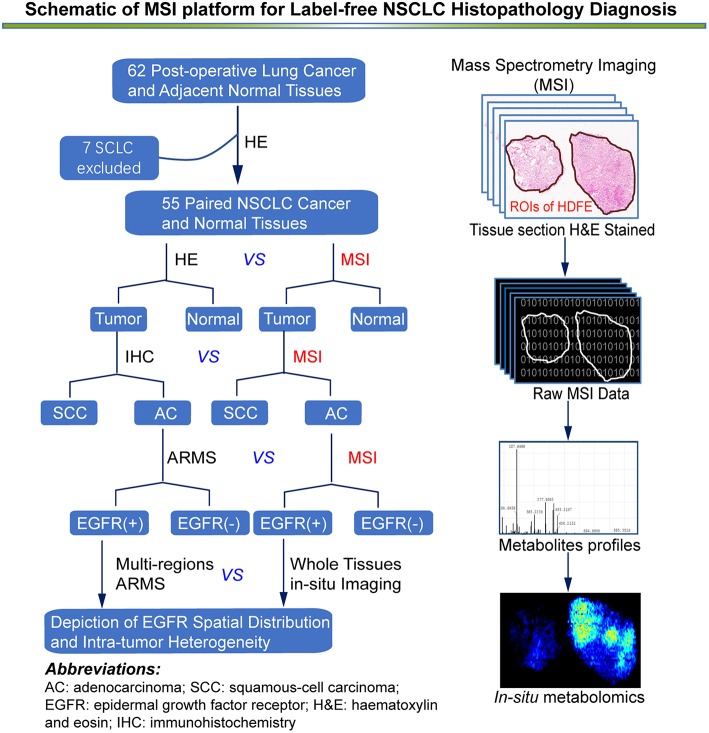
An overview of MSI platform for label-free NSCLC pathology diagnosis. Definition of regions of interest (ROIs) and strategy for developing an *in situ* metabolomics method to discover reliable diagnostic biomarkers to classify molecular pathology and map EGFR mutation status in NSCLC tissues.

All LADC tissue sections were subjected to AFADESI-MSI analysis and we constructed three diagnostic model: tumor vs. normal, AC vs. SCC and *EGFR*-mutated-positive vs. *EGFR*-wild-type as the flow chart described (Figure 1). Raw mass spectrometry data were extracted from the selected regions of interest based on the visual ion image and the corresponding optical image of the tissue section. The raw dataset matrixes were imported into MarkerView (v1.2.1, AB SCIEX) for background deduction, peak picking, and peak alignment. Filtered quality control data were then imported into SIMCA-P software (v14.1, Umetrics AB, Umea, Sweden) to conduct orthogonal projections to latent structures discriminant analysis (OPLS-DA), then the OPLS-DA was applied for the exploration of discriminating variables of three diagnostic models, respectively (29). Discriminating variables with both high covariance and correlation were preferentially selected by S-plot and variable importance (26, 28). Biomarker candidates were further confirmed by an independent *t* test (Microsoft Office Excel 2010), and the variation in and comparison of potential biomarker levels between the experimental groups were presented as histograms (GraphPad Prism 6.02). The receiver operating characteristic and area under the curve values were used to assess the diagnostic power of the selected variables using SPSS (v19.0.0).

## Results

A complete description of the specimens used in this study, including information regarding number, sex, age, type, Eastern Cooperative Oncology Group performance status scores, smoking history, tumor-node-metastasis stage, and *EGFR* status, is provided in Table 1 and Table S1. Representative mass spectra from human lung cancer tissue and corresponding adjacent normal tissue, as well as different pathology types and the *EGFR* mutation status of lung cancer acquired by AFADESI-MSI, are provided in Figures S1–S3 and Table 2. More detailed information on these three diagnostic models using MSI is described below.

**Table 1:** Summary of the lung cancer specimens used in this study (*N* = 55).

**Median age, years (range)**	62 (33–79)
Sex, no. (%)	
Men	37 (67.2)
Women	18 (33.8)
**Smoking status, no. (%)**	
Current smoker	20 (36.4)
Former smoker	8 (14.5)
Never smoker	25 (45.5)
Unknown	2 (3.6)
**Histopathology, no. (%)**	
AC	27 (49.1)
EGFR 19 exon del	6 (10.9)
EGFR 21 exon L858R	4 (7.2)
EGFR wild	17 (30.9)
SCC	28 (50.9)
**Stage^a^, no. (%)**	
I and II	36 (65.4)
IIIA	11 (20)
IIIB	4 (7.3)
IV	4 (7.3)

a *TNM staging is evaluated according to the International Association for the Study of Lung Cancer (IASLC) 7th edition*.

**Table 2:** Potential biomarkers of lung tumors and their tentative identifications results.

**No**.	***m/z***	**Adduct ion**	**Postulated elemental composition**	**MS/MS data**	**Potential results**	**AUC**
1	99.0091	[M-H]^−^	C_4_H_4_O_3_	99,72	Succinic anhydride	0.751
2	104.0333	[M-H]^−^	C_3_H_7_NO_3_	104,74,72	Serine	0.690
3	151.0244	[M-H]^−^	C_5_H_4_N_4_O_2_	151,108	Xanthine	0.772
4	206.0537	[M+Na]^+^	C_5_H_14_NO_4_PNa^+^	206,146,86	[Phosphorylcholine+Na]^+^	0.726
5	222.0272	[M+K]^+^	C_5_H_14_NO_4_PK^+^	222,162,104,86	[Phosphorylcholine+K]^+^	0.687
6	251.2015	[M-H]^−^	C_16_H_28_O_2_	251,80,59	FA(16:2)^a^	0.740
7	301.2166	[M-H]^−^	C_20_H_30_O_2_	301,257,203,59	FA(20:5)	0.700
8	305.2482	[M-H]^−^	C_20_H_34_O_2_	305,249,59	FA(20:3)	0.612
9	307.2630	[M-H]^−^	C_20_H_36_O_2_	307,263,59	FA(20:2)	0.800
10	327.2317	[M-H]^−^	C_22_H_32_O_2_	327,283,59	FA(22:6)	0.852
11	329.2480	[M-H]^−^	C_22_H_34_O_2_	329,285,59	FA(22:5)	0.881
12	331.2641	[M-H]^−^	C_22_H_36_O_2_	331,313,287,59	FA(22:4)	0.772
13	335.2964	[M-H]^−^	C_22_H_40_O_2_	335,59	FA(22:2)	0.813
14	359.2951	[M-H]^−^	C_24_H_40_O_2_	359,59	FA(24:4)	0.867
15	436.2833	[M-H]^−^	C_21_H_44_NO_6_P	436,239,196,140,78	PE(16:0/0:0)^a^	0.612
16	462.3007	[M-H]^−^	C_23_H_46_NO_6_P	462,265,196,140,78	PE(18:2/0:0)	0.687
17	464.3141	[M-H]^−^	C_23_H_48_NO_6_P	464,462,196, 78	PE(18:2/0:0)	0.810
18	758.5699	[M+H]^+^	C_42_H_81_NO_8_P^+^	575,502,496,478,184,86	PC(16:0/18:2)	0.793
19	760.5856	[M+H]^+^	C_42_H_82_NO_8_P	760,496,184	PC(16:0/18:1)^a^	0.824
20	782.5666	[M+Na]^+^	C_42_H_82_NO_8_PNa^+^	782,184	[PC(34:1)+Na]^+^	0.864
21	784.5832	[M+Na]^+^	C_42_H_84_NO_8_PNa^+^	784, 184, 86	[PC(34:0)+Na]^+^	0.725

a *(X:Y) represents the number of carbon atoms (X) and the number of double bonds (Y) in the fatty acid chains*.

*PC, glycerophosphocholine; PE, glycerophosphoethanolamines; FA, fatty acid; AUC, area under the curve*.

### Lipid Profiles Facilitate Accurate Classification of Tumor

**Figure 2:**
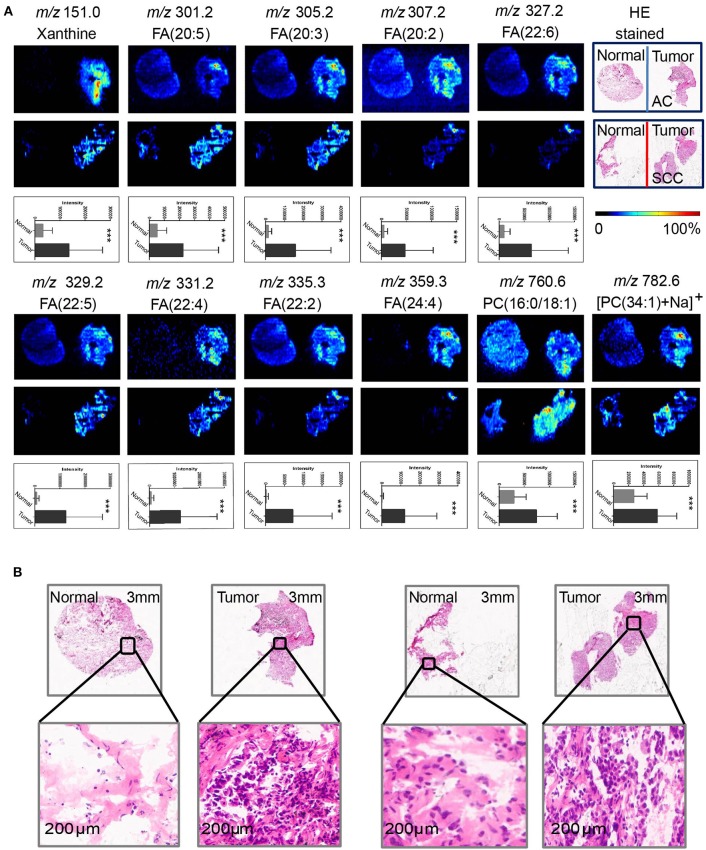
Distributions of representative potential biomarkers (group) across tumors and adjacent normal tissue sections from AC and SCC lung cancer. **(A)** Xanthine; FA(20:5); FA(20:3); FA(20:2); FA(22:6); FA(22:5); FA(22:4); FA(22:2); FA(24:4); PC(16:0/18:1); [PC(34:1)+Na]^+^]. **(B)** Optical images of corresponding H&E stained sections and the amplified figures of tumor and normal lung tissues (× 200).

AFADESI-MSI analysis was performed on 55 paired, treatment-naïve, post-operative lung cancer tissue sections, and corresponding normal tissues to discover potential diagnostic biomarkers for use in differentiating malignant tumors and normal tissue (Figure S5A) according to the protocol outlined in our previous work (20). The most prevalent metabolites species observed in lung cancer tissues were glycerophosphocholines (PCs) and fatty acids (FAs). Eleven metabolites were discovered to be specially enriched in NSCLC tumor tissues, with the signal acquired from tumor tissues being stronger than that obtained from adjacent normal tissues. Both potential biomarkers showed a clear distinction between tumor tissues and normal tissues and could depict a clear tissue contour using MSI shown in Figure 2. The spatial distributions of these metabolites in the ion images were consistent with the statistical trends shown in the histogram. In addition, these differential metabolites group achieved a high diagnostic power of 0.991 after binary logistic regression (Figures S4A,D) for classification of tumor and normal tissues.

### Lipid Profiles Facilitate Label-Free Lung Cancer Molecular Diagnosis and *EGFR* Mutations Detection

**Figure 3:**
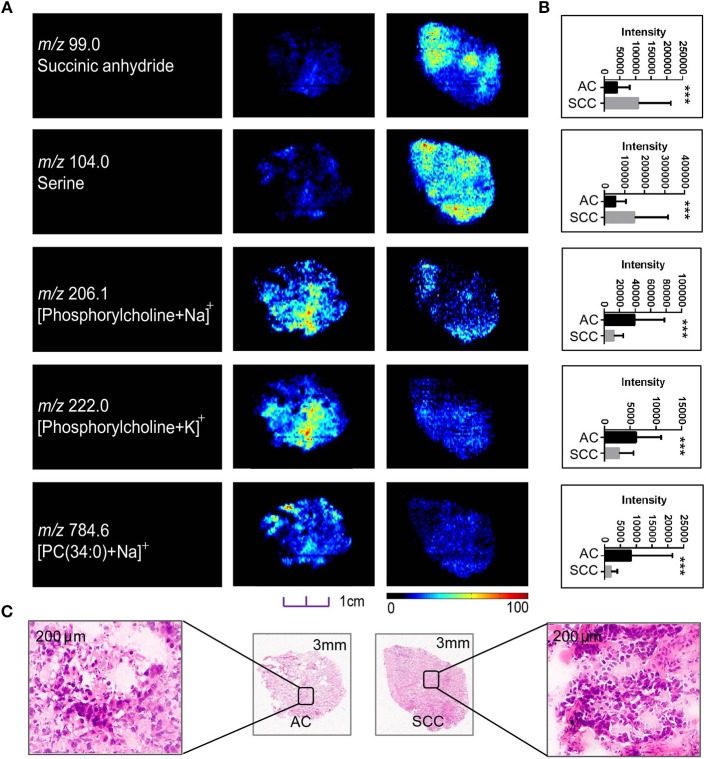
Distributions of representative potential pathology-related biomarkers **(A)** across AC and SCC tumors and the histogram **(B)** showed relative expression level of these biomarkers in AC (black box) and SCC tumors (gray box). **(C)** Optical images of corresponding H&E stained sections the amplified figures (× 200). PC, Phosphorylcholine. *p < 0.05, **p < 0.01, ***p <0.001 (*t*-test).

**Figure 4:**
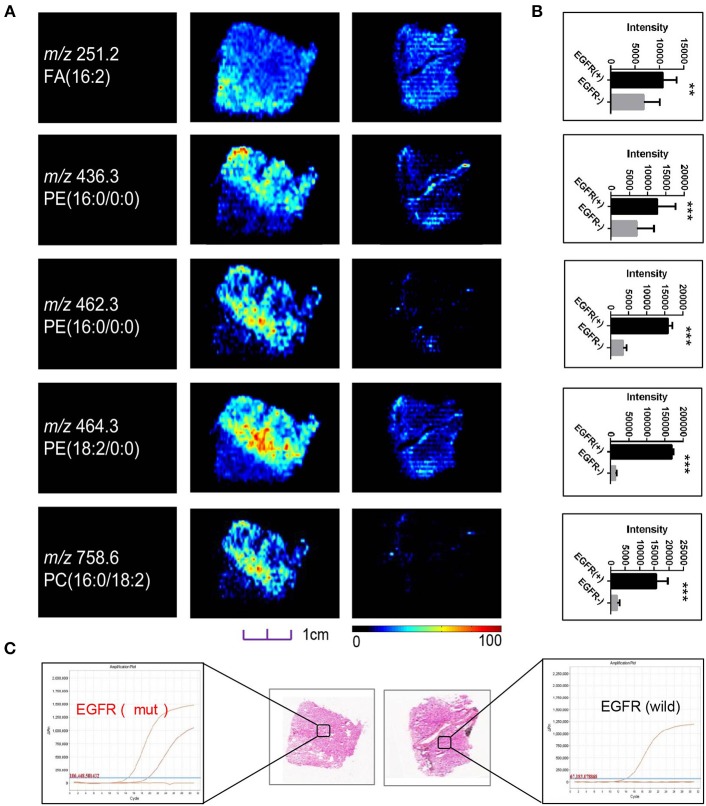
**(A)** Distributions of representative potential EGFR-related biomarkers **(A)** across different EGFR mutation status of lung cancer, and histogram **(B)** the boxplot showed relative expression level of these biomarkers in *EGFR*-mutated-positive (gray box) and *EGFR*-wild (black box) samples. **(C)** Optical images of corresponding H&E stained sections and DNA amplification plot of *EGFR* by ARMS; *p < 0.05, **p < 0.01, ***p <0.001 (*t*-test).

**Figure 5:**
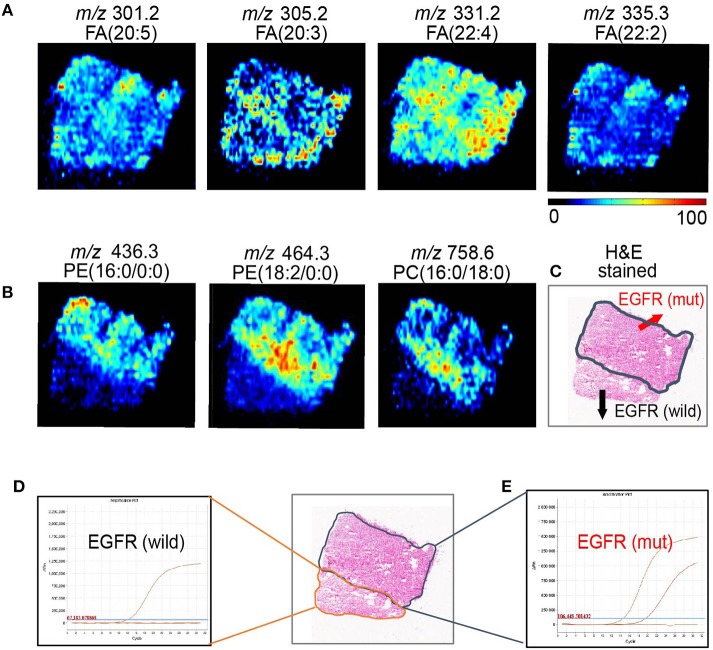
AFADESI-MSI revealed the spatial heterogeneity of *EGFR* by *in situ* metabolomics imaging. **(A)** The tumor-related biomarkers of FA(20:5), FA(20:3), FA(22:4), FA(22:2) were used to showcase the overall contour of cancerous tissue. **(B)** The *EGFR*-related biomarkers of PE(16:0/0:0), PE(18:2/0:0), and PE(16:0/18:0) present the *EGFR* mutation spatial distribution heterogeneity. **(C)** With region of tissue harbor *EGFR* mutation delineated by dotted blue line shown in optical image. DNA amplification plot of *EGFR* by ARMS. **(D)** *EGFR* mutant-wild; **(E)** *EGFR* mutant positive.

Although sharing most of the malignancy signature, different pathological types of lung cancer were characterized by differences in their metabolic behavior (30, 31). We further investigated the metabolic difference of AC from SCC and tried to explore discriminative biomarkers to classify these two groups (Figure S5B). Interestingly, MSI images (Figure 3) showed that the phosphorylcholine molecules and PC had a stronger ions intensity in AC compared with SCC tissues while the SCC tissues were specially enriched with succinic anhydride and serine. These biomarkers group generated by MSI achieved a diagnostic sensitivity of 85.2% (23/27) and 82.1% (23/28) for AC and SCC, respectively, with a high diagnostic power of 0.827 for pathological diagnosis model (Figures S4B,D). As the MSI method could intuitively reveal the discriminative biomarkers distribution in tumor tissues without antibody staining, the difference of metabolites abundance in tumor tissues could be used to study the tumor behavior characteristic between tumor subtypes. Hence, we further validated the ability of the MSI method to distinguish *EGFR-*mutated-positive from *EGFR*-wild-type AC samples. There was a trend indicating clear separation between *EGFR-*mutated-positive and *EGFR*-wild-type samples after multivariate statistical analysis (Figure S5C), whereas *EGFR-*mutated-positive subtypes (19-DEL vs. 21-L858R) were not distinguished well (Figure S5D), possibly due to the number of specimens. We also discovered a panel of lipids associated with *EGFR* mutations status. As shown in Figure 4, the phospholipids molecules *m/z* 436.3 [PE(16:0/0:0)], *m/z* 462.3 [PE(18:2/0:0)], *m/z* 464.3 [PE(18:2/0:0)], and *m/z* 758.6 [PC(16:0/18:2)] were discovered to be specially enriched in *EGFR-*mutated-positive compared with *EGFR*-wild-type samples. These phospholipids biomarkers achieved a diagnostic sensitivity of 82.3% (14/17) and 80% (8/10) for these two groups with a high diagnostic power of 0.880 (Figures S4C,D). Noteworthy, in one AC sample (N34, EGFR-mutated-positive), we observed that these phospholipids had a distinct distribution in separate regions of the same tumor tissue. We deduce that the heterogeneous distribution of phospholipids in tumor tissues may be related to the spatial distribution heterogeneity of *EGFR* mutations. To validate this hypothesis, we confirmed that the N34 sample had a high abundance of tumor cells according to imaging of tumor-related biomarkers (Figure 5A). Then, we extracted nucleic acids from different regions of tumor tissue for ARMS analysis, which revealed that only the region providing the strongest ion intensities of phospholipids (Figure 5B) for the *EGFR* mutation returned a positive result (Figures 5C–E). These results suggested that phospholipids molecules, including PC and PE, but not FA, might be more reliable biomarkers to characterize *EGFR* mutation status.

## Discussion

Endogenous metabolites serve as direct signatures of biochemical activity and downstream products of gene expression, and are therefore easier to correlate with phenotype and can help to understand the pathogenesis (31). In this study, we discovered several reliable discriminative tumor biomarkers which could be used for classification of tumor and normal samples. Based on the tumor associated lipids imaging, tumor margin and contour could be immediately recognized and mapped, which may benefit accurate surgical resections. We also found the phospholipids were specially enriched in AC compared with SCC tissues. Noteworthy, with the higher ions intensity of phospholipids in *EGFR-*mutated-positive AC compared with *EGFR-*wild-type samples. These findings were consistent with the result that abnormal choline phospholipid metabolism is a hallmark of cancer (32). Two independent studies also revealed *EGFR*-mutated-positive NSCLC had a unique metabotype according to lipidomic profiling in lung pleural effusion (33) and phospholipids of tumor extracellular vesicles were different in gefitinib-resistant NSCLC cells from gefitinib-sensitive NSCLC cells (34). These results along with our observation showed phospholipids may represent reliable discriminative biomarkers associated with *EGFR* mutations status.

Tumor tissues are known to contain both tumor cells and normal cells, with transitional and apoptotic cells also included, the *EGFR* mutations in separate regions of the same tumor tissue may be highly heterogeneous. In addition, *EGFR* mutations spatial heterogeneity remains one of most important reason leading to targeted drug resistance, which has posed a series of challenge to both accurate diagnosis and personalized therapy. The routine *EGFR* mutations detection methods inherently lose all spatial information during pre-treatment process, may obscure the difference between separate spatial regions of tumor tissues. Hence, current gene mutations detection methods provided little information of *EGFR* mutations spatial distribution, were unable to assess the intra-tumor heterogeneity. For a more accurate *EGFR* mutations diagnosis, several research groups (12–14) tried to apply laser capture micro-dissection to extract tumor materials in different tumor spatial regions and perform multiregional genomic sequencing to generate a comprehensive *EGFR* mutations spatial landscape. In addition, isotopically labeled *EGFR*-TKIs probes (35, 36) have also been used for *in vivo* molecular imaging of *EGFR* spatial distribution in lung tumor xenografts. However, these methods have inherent limitations: a large amount of tumor tissue is needed, the sample pre-treatment is complex and time consuming. These methods remain available only in a low-throughput manner, and therefore cannot be routinely applied clinically for lung cancer diagnosis.

As MSI can directly map the spatial distribution of molecules of interest in association with pathological features in small amount of tissue sample with minimal pre-treatment, has been recognized as a label-free IHC form (25). The matrix assisted laser desorption/ionization mass spectrometry imaging (MALDI-MSI) technique has been used to explore the proteomic differences and intuitively reveal HER2 receptor status in breast cancer (37). In this study, we also revealed that the phospholipids signature was able to accurately classify *EGFR* mutation status. According to phospholipids imaging, the *EGFR* mutation spatial distribution map in whole tumor tissues was not difficult to generate, thereby we could visually observe the *EGFR* mutations spatial distribution features. We also performed multi-regions ARMS *EGFR* detection in tissue section of each tumor sample. Interestingly, we found that only the regions providing the strongest ions intensity of phospholipids were *EGFR*-mutated-positive, suggested that phospholipids imaging could reveal *EGFR* mutations spatial distribution heterogeneity. To our knowledge, this is the first time to generate a comprehensive *EGFR* spatial distribution landscape and observe *EGFR* mutation intratumor heterogeneity based on metabolites imaging. The MSI method may provide a new perspective to analyze and monitor the *EGFR* mutation status *in vivo*, potentially benefitting real-time monitoring of targeted therapy.

As this is a preliminary study, the number of cases studied here was limited, our findings of the phospholipid biomarkers for the identification of *EGFR* mutations status need to be validated in a larger cohort of patients. Second, whether or not these biomarkers can predict the clinical response of *EGFR-*targeting drugs remains unclear. Future studies to use these phospholipid biomarkers for dynamic observation of *EGFR*-TKIs efficacy are currently under consideration as a possible extension of our work.

## Data Availability

The raw data supporting the conclusions of this manuscript is available in Data Sheets S1 and S2.

## Ethics Statement

This study was carried out in accordance with the recommendations of the ethical review community of the Fifth Medical Center of Chinese PLA General Hospital with written informed consent from all subjects. All subjects gave written informed consent in accordance with the Declaration of Helsinki. The protocol was approved by the ethical review community of the Fifth Medical Center of Chinese PLA General Hospital.

## Author Contributions

XLiu and ZA designed and supervised the entire study. MZ, TL, and JH planned the experiments. HX, XLi, FY, JW, and QM provided lung cancer samples, clinical diagnosis information, and confirmed tumor margin and content. HH performed ARMS analysis of EGFR. BL and CT had participated in the discussion. MZ, TL, and JH performed AFADESI-MSI analysis, data mining and processing, and wrote the manuscript.

### Conflict of Interest Statement

The authors declare that the research was conducted in the absence of any commercial or financial relationships that could be construed as a potential conflict of interest.

## Supplementary Material

Table S1 Information on human specimens used in this study.

Data Sheet S1 Raw mass spectrometry expression matrix in positive ions model.

Data Sheet S2 Raw mass spectrometry expression matrix in negative ions model.

Figure S1 Representative mass spectra from human lung cancer tissue and corresponding adjacent normal tissue acquired by AFADESI-MSI.

Figure S2 Representative mass spectra from AC and SCC tumors samples acquired by AFADESI-MSI.

Figure S3 Representative mass spectra from EGFR-mutated-positive samples and EGFR-wild-type samples acquired by AFADESI-MSI.

Figure S4 The ROC curve illustrates the combined discriminatory performance of a group of valuable clinical biomarkers in **(A)** Tumor diagnosis model, **(B)** pathology type classification model, **(C)** EGFR mutation detection model, and **(D)** the histogram revealed the diagnostic power of each discriminatory biomarker.

Figure S5 Score plot of the OPLS-DA models derived from AFADESI-MSI data for **(A)** tumor diagnosis model, **(B)** pathology type classification model, **(C)** EGFR mutation detection model, and **(D)** EGFR mutation subtypes detection model.

## Abbreviations

AC: adenocarcinoma
SCC: squamous-cell carcinoma
AFADESI: air-flow-assisted desorption electrospray ionization
ARMS: amplification refractory mutation system
EGFR: epidermal growth factor receptor
H&E: haematoxylin and eosin
LC-MS: liquid chromatography mass spectrometry
MS/MS: tandem mass spectrometry
MSI: mass spectrometry imaging
NSCLC: non-small-cell lung cancer
SCLC: small cell lung cancer
TKI: tyrosine kinase inhibitor.
