# Supplementary material for: Accurate Classification of Non-small Cell Lung Cancer (NSCLC) Pathology and Mapping of EGFR Mutation Spatial Distribution by Ambient Mass Spectrometry Imaging
Source: Front Oncol. 2019 Aug 28;9:804. doi: 10.3389/fonc.2019.00804 (PMC6722907; doi:10.3389/fonc.2019.00804)
Supplement: Table S1 — Information on human specimens used in this study. [file Table_1.DOCX]

**Table S1**; Information on human specimens used in this study.

| **ID** | **Sex** | **Age**  **(year)** | **Smoking**  **(year)** | **Pathology** | **TNM** | **Stage** | **ECOG Ps score** | **EGFR** |
| --- | --- | --- | --- | --- | --- | --- | --- | --- |
| N1 | Male | 79 | 40 | SCC | T2N0M0 | IB | 1 | n.a. |
| N2 | Male | 60 | 40 | AC | T2N0M0 | IB | 1 | wild |
| N3 | Male | 52 | 20 | AC | T2N0M0 | IB | 1 | wild |
| N4 | Female | 61 | 0 | AC | T2N0M0 | IB | 2 | wild |
| N5 | Male | 69 | 0 | AC | T2N2M0 | IIIA | 1 | 21-L858R |
| N6 | Female | 33 | 0 | SCC | T2N1M0 | IIB | 1 | n.a. |
| N7 | Male | 39 | 20 | SCC | T3N1M0 | IIIA | 1 | n.a. |
| N8 | Female | 40 | 0 | SCC | T4N2M0 | IIIB | 1 | n.a. |
| N9 | Female | 66 | 0 | AC | T1N2M0 | IIIA | 1 | wild |
| N10 | Male | 56 | 20 | SCC | T2N1M0 | IIB | 1 | n.a. |
| N11 | Male | 60 | 40 | AC | T3N2M0 | IIIB | 1 | wild |
| N12 | Female | 75 | 0 | AC | T2N0M0 | IB | 2 | 21L858R |
| N13 | Male | 40 | 20 | SCC | T2N1M0 | IIB | 1 | n.a |
| N14 | Male | 44 | 20 | SCC | T3N2M1 | IV | 1 | n.a. |
| N15 | Male | 58 | 30 | AC | T3N0M0 | IIB | 1 | wild |
| N16 | Female | 45 | 0 | SCC | T2N1M0 | IIB | 1 | n.a |
| N17 | Male | 50 | 30 | SCC | T2N1M0 | IIB | 1 | n.a. |
| N18 | Female | 62 | 0 | SCC | T2N1M0 | IIB | 1 | n.a. |
| N19 | Male | 64 | 30 | AC | T2N0M0 | IB | 1 | wild |
| N20 | Male | 62 | 20 | SCC | T2N1M0 | IIB | 1 | n.a. |
| N21 | Male | 65 | 40 | SCC | T3N0M0 | IIB | 1 | n.a. |
| N22 | Male | 69 | 0 | SCC | T2N2M0 | IIIA | 1 | n.a. |
| N23 | Female | 66 | 0 | AC | T1N0M0 | IA | 1 | 19-DEL |
| N24 | Male | 63 | 40 | SCC | T2N1M0 | IIB | 1 | n.a. |
| N25 | Male | 50 | 0 | AC | T1N2M0 | IIIA | 1 | wild |
| N26 | Female | 60 | 0 | SCC | T2N2M0 | IIIA | 1 | n.a. |
| N27 | Female | 46 | 0 | AC | T4N2M1 | IV | 1 | 19-DEL |
| N28 | Male | 66 | 30 | AC | T2N1M0 | IIB | 1 | wild |
| N29 | Male | 79 | 10 | SCC | T2N0M0 | IB | 2 | n.a. |
| N30 | Female | 67 | 0 | AC | T2N1M0 | IIB | 2 | 19-DEL |
| N31 | Male | 74 | 20 | SCC | T1N1M0 | IIB | 1 | n.a. |
| N32 | Female | 62 | 0 | AC | T2N1M0 | IIB | 1 | wild |
| N33 | Male | 69 | 20 | AC | T2N0M0 | IB | 1 | wild |
| N34 | Male | 50 | 20 | AC | T2N2M0 | IIIA | 1 | 21-L858R |
| N35 | Female | 41 | 0 | AC | T1bN2M1 | IV | 2 | wild |
| N36 | Female | 73 | 0 | AC | T2N1M0 | IIB | 2 | 19-DEL |
| N37 | Male | 77 | 0 | SCC | T2aN1M0 | IIB | 1 | n.a. |
| N38 | Female | 60 | 0 | AC | T2bN0M0 | IIA | 1 | wild |
| N39 | Male | 63 | 30 | SCC | T1bN0M0 | IA | 1 | n.a. |
| N40 | Male | 71 | 30 | SCC | T2aN2M0 | IIIA | 1 | n.a. |
| N41 | Female | 70 | 0 | AC | T3aN0M0 | IIB | 2 | 19-DEL |
| N42 | Male | 41 | 20 | SCC | T2bN2M1 | IV | 1 | n.a. |
| N43 | Male | 65 | 50 | SCC | T2bN2M0 | IIIA | 1 | n.a. |
| N44 | Male | 49 | 20 | SCC | T4N1M0 | IIIA | 1 | n.a. |
| N45 | Male | 58 | 0 | SCC | T3N0Mx | IIB | 2 | n.a. |
| N46 | Male | 59 | 30 | SCC | T1bN2M0 | IIIA | 1 | n.a. |
| N47 | Male | 64 | 0 | SCC | T2aN1M0 | IIB | 1 | n.a. |
| N48 | Male | 73 | 30 | AC | T2aN0M0 | IB | 1 | wild |
| N49 | Male | 72 | 30 | SCC | T1bN0M0 | IA | 2 | n.a. |
| N50 | Female | 59 | 0 | AC | T2aN0M0 | IB | 1 | wild |
| N51 | Female | 57 | 0 | AC | T1bN0M0 | IA | 1 | 21-L858R |
| N52 | Male | 65 | 20 | SCC | T3N2M0 | IIIB | 1 | n.a. |
| N53 | Male | 74 | 0 | AC | T2bN0M0 | IIA | 1 | wild |
| N54 | Male | 68 | 30 | AC | T2aN0M0 | IB | 2 | 19-DEL |
| N55 | Male | 75 | 20 | AC | T3N2M0 | IIIB | 1 | wild |
| Abbreviations: AC, Adenocarcinoma; SCC, Squamous cell carcinoma; lung cancer TNM staging is evaluated according to International Association for the Study of Lung Cancer (IASLC) 7^th^ edition; n.a.=not assessed; ECOG, Eastern Cooperative Oncology Group; PS, performance status | | | | | | | | |
