# Supplementary figures and images for: Accurate Classification of Non-small Cell Lung Cancer (NSCLC) Pathology and Mapping of EGFR Mutation Spatial Distribution by Ambient Mass Spectrometry Imaging
Source: Front Oncol. 2019 Aug 28;9:804. doi: 10.3389/fonc.2019.00804 (PMC6722907; doi:10.3389/fonc.2019.00804)

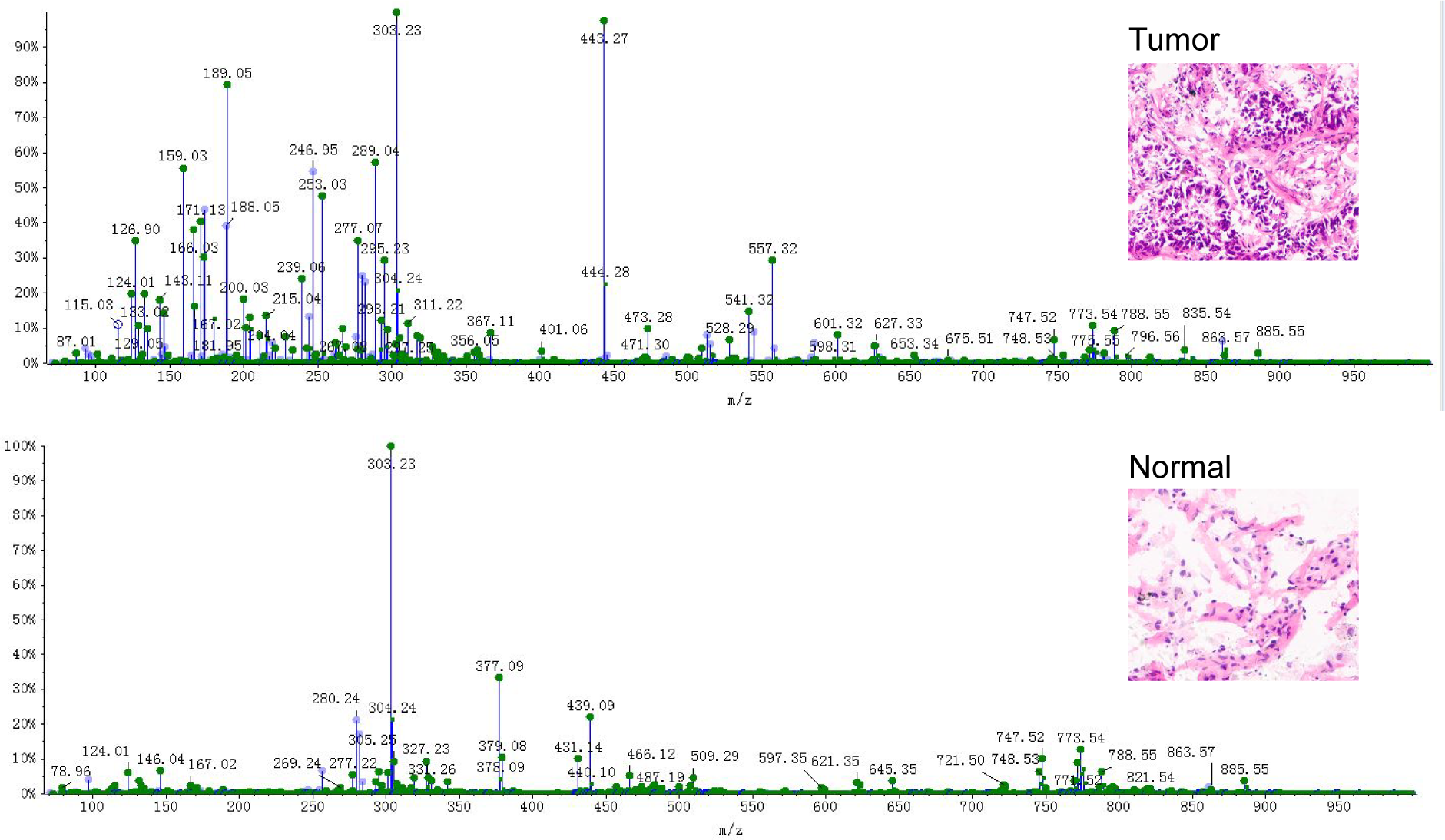

Supplement: Figure S1 — Representative mass spectra from human lung cancer tissue and corresponding adjacent normal tissue acquired by AFADESI-MSI. [file Image_1.TIF]

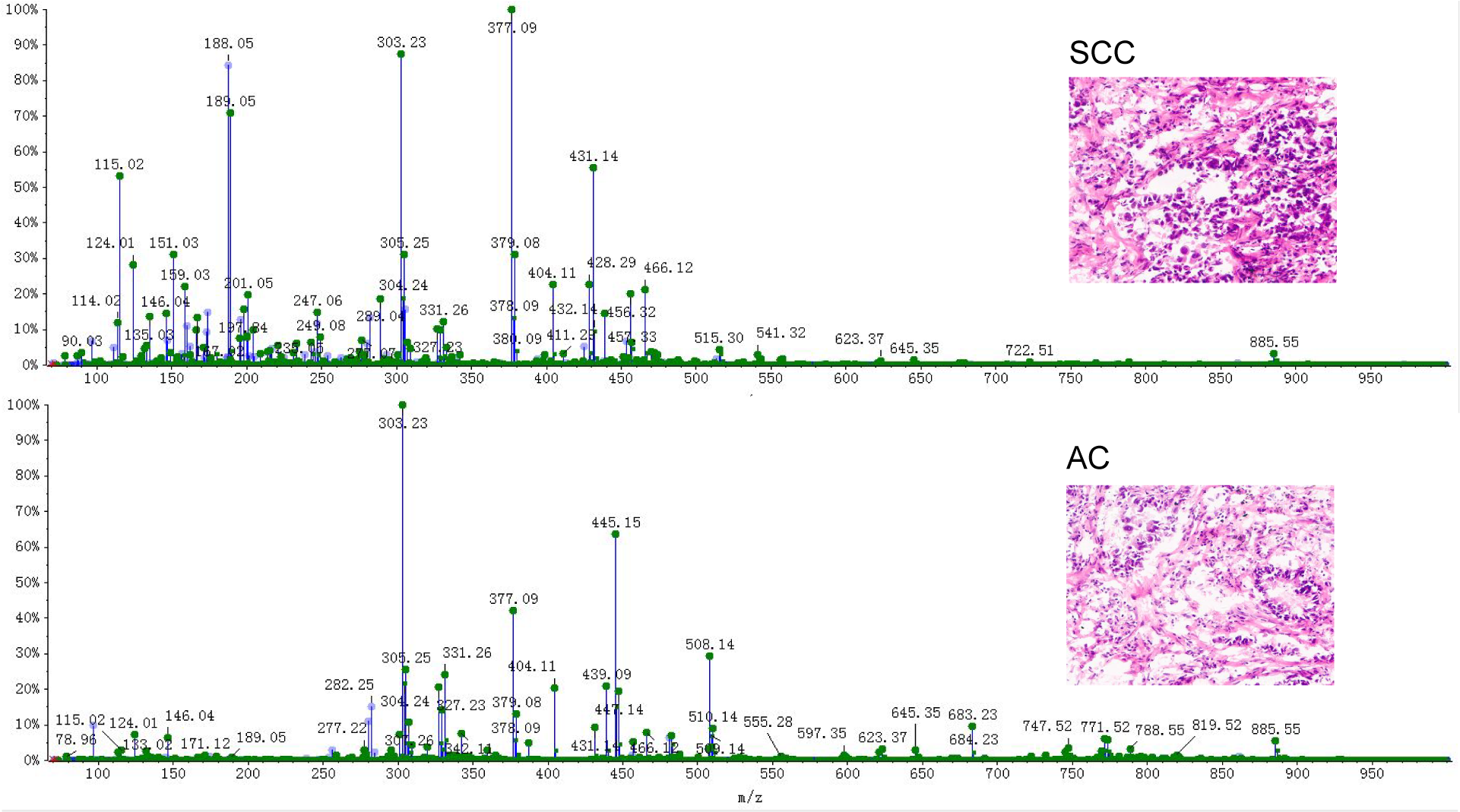

Supplement: Figure S2 — Representative mass spectra from AC and SCC tumors samples acquired by AFADESI-MSI. [file Image_2.TIF]

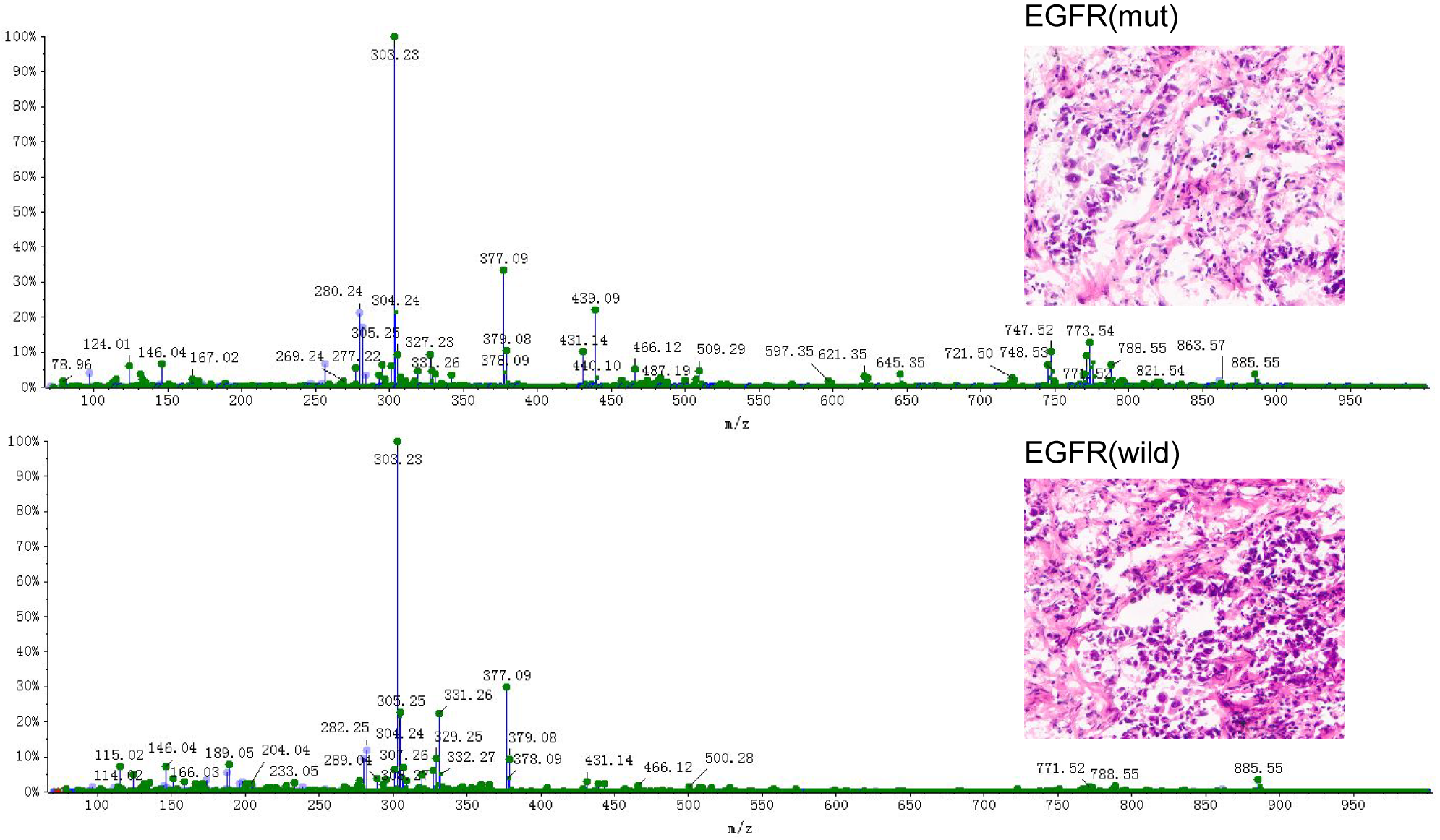

Supplement: Figure S3 — Representative mass spectra from EGFR-mutated-positive samples and EGFR-wild-type samples acquired by AFADESI-MSI. [file Image_3.TIF]

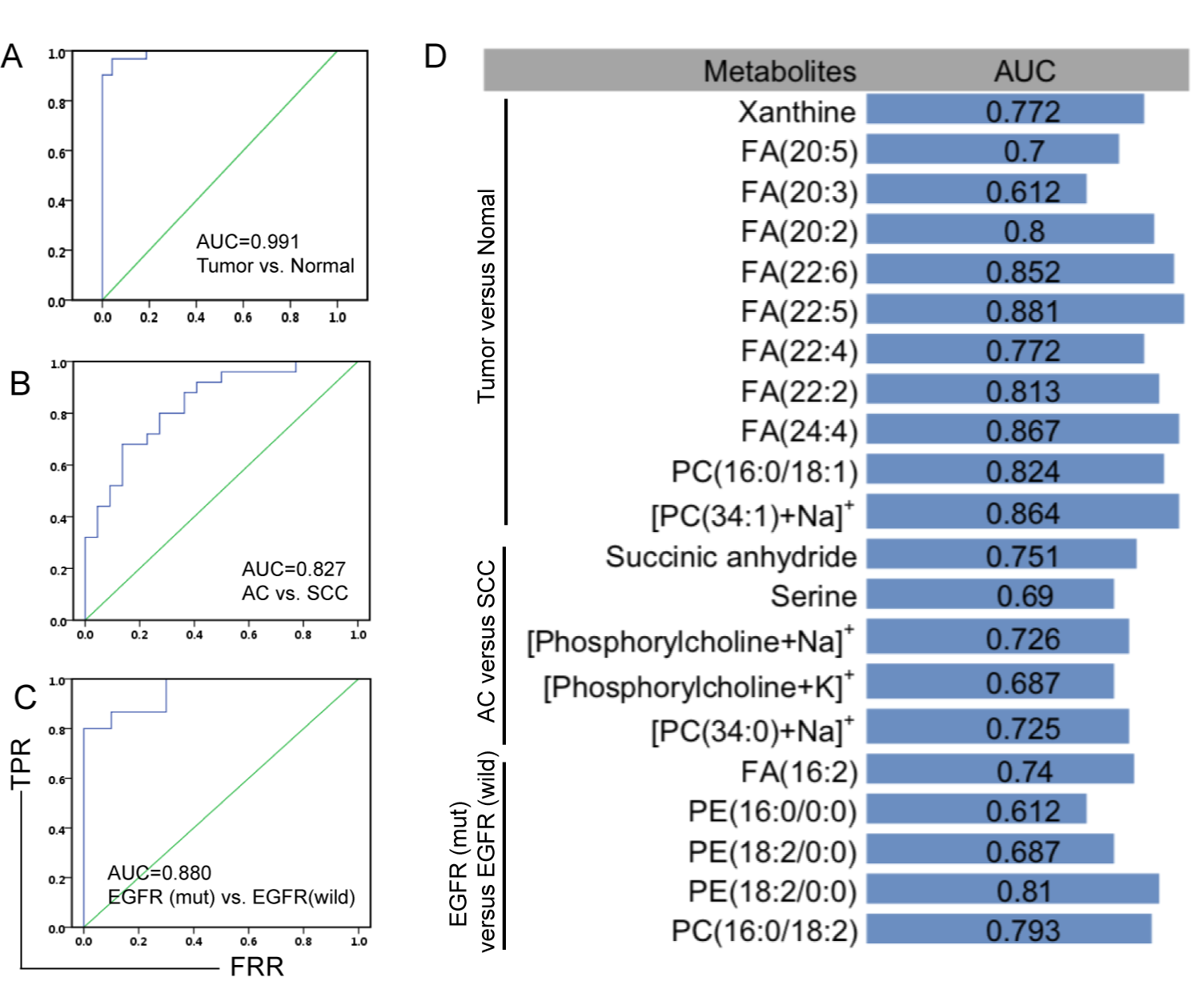

Supplement: Figure S4 — The ROC curve illustrates the combined discriminatory performance of a group of valuable clinical biomarkers in (A) Tumor diagnosis model, (B) pathology type classification model, (C) EGFR mutation detection model, and (D) the histogram revealed the diagnostic power of each discriminatory biomarker. [file Image_4.TIF]

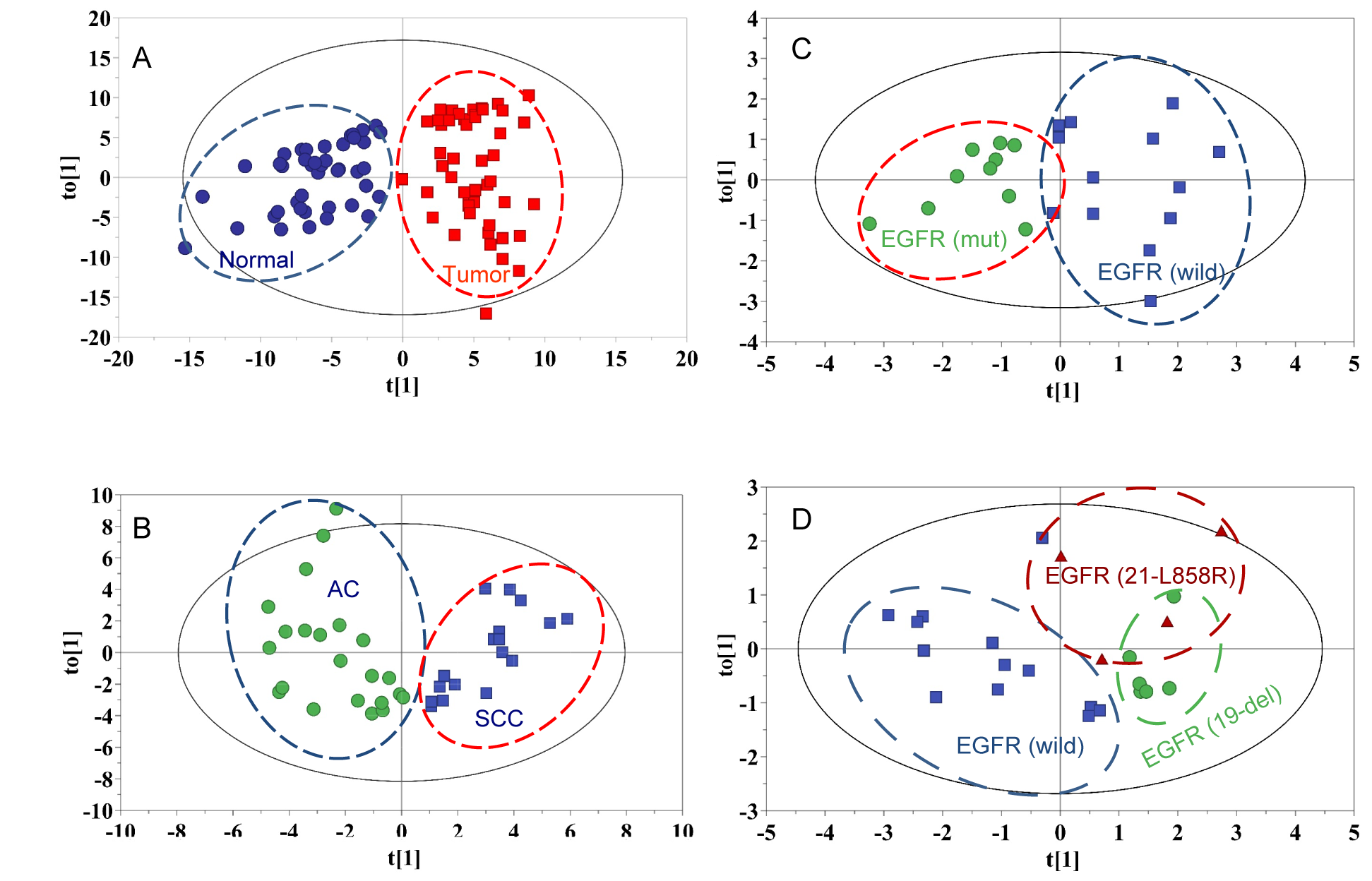

Supplement: Figure S5 — Score plot of the OPLS-DA models derived from AFADESI-MSI data for (A) tumor diagnosis model, (B) pathology type classification model, (C) EGFR mutation detection model, and (D) EGFR mutation subtypes detection model. [file Image_5.TIF]
